# An Investigation on the Effects of Partial Replacement of Rapeseed Cake in Ayrshire Dairy Heifers’ Diets with By-Product Source of Animal Protein on Body Weight Dynamics, Nutrient Balancing, and Blood Biochemical Markers

**DOI:** 10.3390/ani13111856

**Published:** 2023-06-02

**Authors:** Nikolai P. Buryakov, Dmitrii E. Aleshin, Maria A. Buryakova, Anastasiya S. Zaikina, Ivan K. Medvedev, Darya A. Zemyachkovskaya, Georgy Y. Laptev, Larisa A. Ilina, Ahmed I. El Sheikh, Mohamed M. Fathala, Ferial M. Sahwan

**Affiliations:** 1 Department of Feeding Animals, Institute of Animal Science and Biology, Russian State Agrarian University—Moscow Timiryazev Agricultural Academy, 49 Timiryazevskaya Str., Moscow 127434, Russia; 2 Department of Physiology, Ecology and Biochemistry of Animals, Institute of Animal Science and Biology, Russian State Agrarian University—Moscow Timiryazev Agricultural Academy, 49 Timiryazevskaya Str., Moscow 127434, Russia; 3 Limited Liability Company A-1 First Genetic Company, Mironovskaya 33, p. 11, Moscow 105187, Russia; 4 Molecular Genetic Laboratory, BIOTROF+ Ltd., Pushkin, Saint-Petersburg 196650, Russia; 5 Department of Large Animal Husbandry, Faculty of Bioengineering and Biotechnology, Saint-Petersburg State Agrarian University, Pushkin, Saint-Petersburg 196601, Russia; 6 Department of Veterinary Public Health, College of Veterinary Medicine, King Faisal University, Al-Hofuf 31982, Saudi Arabia; 7 Animal Husbandry and Wealth Development Department, Faculty of Veterinary Medicine, Alexandria University, Bab Sharqi 5424041, Egypt

**Keywords:** Russian Ayrshire heifers, by-products, protein feeds, growth dynamics, nitrogen balance, biochemical parameters

## Abstract

**Simple Summary:**

According to the latest estimate of the Food and Agriculture Organization of the United Nations, it is expected that by 2050 the world’s population will increase to 10 billion people, which will lead to a great increase in the demand for agricultural products. By this time, the world’s population will need 60–70% more animal products than it consumes today and, consequently, a high demand for feed will be required. Recently, the secondary use of the by-products of the processing industry, obtained as a result of the production of poultry meat, which can be included in the diets of farm animals, has become a popular topic in the feed industry. Russian veterinary legislation allows the use of poultry meat processing waste in the feeding of ruminants, which is consistent with EU Regulation No. 1069/2009 of 21 October 2009, which states: “Materials obtained from animals should not be fed to animals belonging to the species from which they were obtained”. Our research is developed to use a new feed (concentrate) from the remains of non-edible wastes (blood, heads, legs and internal non-edibles) together with plant proteins (white lupine grain) in feeding young cattle of the Russian Ayrshire cattle. This article describes the chemical composition of protein concentrate as an alternative feed in the calves’ diet and its effect on the bacterial microbiome, nutrient digestibility and biochemical parameters of calves and heifers.

**Abstract:**

Recently, the secondary use of by-products of the processing industry resulting from the production of poultry meat, which can be included in animal diets, has become a popular topic in the feed industry. For studying the effects of partial replacement of rapeseed cake (RC) with the by-product source of animal protein concentrate Agro-Matic (PCAM) on growth dynamics, nutrient absorption and nitrogen balance, as well as blood biochemical parameters during the growing period, a total of 48 Russian Ayrshire dairy heifers were selected for this experiment and they were divided into three experimental groups (16 in each group, including the control group). The heifers of the control group were fed the basal diet which contained rapeseed cake (30%), while the second (RC + PCAM) and third groups (PCAM + RC) were fed the basal diet after replacing a part of the rapeseed cake with 2.25% and 4.5% of protein concentrate Agro-Matic respectively. The results showed that the weight of heifers treated with PCAM at 3 months of age exceeded the control by 2.3 kg (*p* > 0.05) in group 2 by 4.4 kg (*p* < 0.05). Similar results were obtained at the age of 6 months of raising. Feeding 4.50% protein concentrate Agro-Matic has a positive effect on the digestibility of nutrients; in particular, there was a significant increase in the digestibility of crude protein in the PCAM + RC group (77.23 vs. 73.42%) compared with the control group. Moreover, a similar trend was found in the digestibility of nitrogen in the diet. At the age of 3 months, heifers showed a significant decrease in the concentration of ketone bodies in the second group (1.82 vs. 2.20 mmol/L) relative to the control group. Urea was significantly lower in the RC + PCAM group (5.05 vs. 6.62 mmol/L) relative to the PCAM + RC group, while acid capacity (alkaline reserve) was higher by 2.41% (*p* < 0.05) relative to the control. In the 10th month of age, a positive effect on the blood of heifers was observed, as in the second and the third experimental groups, β-globulin and phosphorus increased (*p* < 0.05), while in the second group aspartate aminotransferase decreased (*p* < 0.05). Consequently, replacing the rapeseed cake with the protein concentrate Agro-Matic revealed an improvement in the dynamics of growth, nutrient digestibility and nitrogen balance, and it has an effect on improving some biochemical parameters of the blood.

## 1. Introduction

Production of poultry by-product, which can be included in other animal diets, has become a popular topic in the feed industry worldwide [1]. Together with meat, bone, blood, feather and fish meal, poultry by-product meal is regarded as one of the important sources of animal protein that can be fed to domestic animals [2,3]. Depending on the substrate being processed, poultry by-product meal’s nutritious value can vary significantly [4]. Considering the vital amino acids, fatty acids, vitamins, and minerals it contains, it is typically a tasty and high-quality feed element. It is highly sought after by the pet food and aquaculture industries in addition to being used in livestock [3]. 

The world of animal husbandry is a priority direction of development due to the greater intensity of development and growth of production [5,6]. Thus, in Russia over the past decade, the production of fish, pig meat, poultry and cattle has increased significantly [6,7]. The main direction of any agricultural production, first of all, is to fully meet the needs of the population for food at the lowest cost of all types of resources, so it is necessary to increase the volume of production, and to improve the assortment and its quality [7,8,9,10,11,12]. In Iran, poultry farm waste amounts to about 12.6 thousand tons per year while, in Turkey, the volume of production at large abattoirs is estimated to have solid by-products of about 1 million tons per year [13,14].

The most urgent task is the introduction of harmless methods of processing biological waste, which at the same time represents a valuable secondary crude material for the production of feed [13,14,15]. It is customary to refer to such waste as non-food waste and low-value food products obtained during the processing of poultry, fish, livestock and other animals [15,16,17]. The total volume of which from the slaughter and evisceration of poultry is at least 26% of the live weight; from the slaughter of pigs, cattle and fish processing it is about 30–35% [13,16,17,18,19,20]. Most by-products can provide organic and inorganic nutrients that are valuable when properly handled and processed [19,20,21,22].

Russian veterinary legislation allows the use of poultry meat processing waste in the feeding of ruminants, which is consistent with EU Regulation No. 1069/2009 of 21 October 2009, which states: “Materials obtained from animals should not be fed to animals belonging to the species from which they were obtained”.

The use of waste from food industries in modern feed production will ensure deep processing of food crude materials of animal and vegetable origin, reduce the cost of production of basic products through the sale of additional products, expand the range of modern feed, develop domestic livestock and poultry farming, and improve the environmental safety of food and processing workshops [7,13,17,22,23].

Organic waste is characterized by a high total solids content of more than 10–15%, which mainly consist of animal proteins and fats [17,24]. Poultry by-products contain approximately 34.2% dry matter, of which 51.8% crude protein, 41.0% fat and 6.3% ash [13]. Solid organic materials contain 32% protein and 54% lipids and have the potential to produce methane from 0.6 to 0.9% in ruminants. Abattoir waste is mostly heterogeneous in composition and atypical, containing a large amount of proteins and fats along with a significant amount of carbohydrates [18,22,23,24,25,26]. Thus, according to Kazemi-Bonchenari et al. (2017), they contain protein (50–63%), essential extract (18–27%) and ash (9–15.5%) [13]. Flour from poultry by-products has the best amino acid composition and can be included up to 10% in the composition of animal feed [15,22,23]. The content of methionine and lysine in the sources of poultry abattoir waste is the same, but the difference in the content of cystine (1.2–1.7%), threonine (1.9–2.2%), arginine (3–3.5%), leucine (3.5–4.1%) and valine (2.8–3.3%) is quite high [13,24].

Ruminants that are reared for the production of a high amount of animal products require a high intake of bypass or slowly degradable protein rich in essential amino acids [27,28]. Protein, which undergoes cleavage in the small intestine, supplies the animal body with essential amino acids for the synthesis of milk and the formation of new body tissues [27,29,30].

In our early works devoted to the use of a new protein feed protein concentrate Agro-Matic (PCAM), which is made from poultry meat processing waste and crushed white lupine grain in feeding lactating cows [31,32,33,34,35], it was shown that the inclusion of it in various quantities in the diet of highly productive Ayrshire dairy cows showed a positive effect on milk productivity and milk quality throughout the lactation period, improved digestibility of nutrients, nitrogen retention in the body without deterioration of the physiological and the sanitary condition of cows. Moreover, the inclusion of 1.5 kg/head/day PCAM was the best regarding economic benefits [31,32,33,34].

Analysis of the rumen content showed that the number of *Bacteroidetes* in cows of groups (1.0 and 1.5 kg/day) receiving protein concentrate in their diets was reduced by 1.5% and 2.9%. There were no significant differences between the control group and the groups receiving PCAM supplements and in most of the rumen microbiota, with the exception of pathogenic microorganisms such as *Peptococcus* and *Fusobacteria*; a significant decrease was noted between the control group and the groups receiving PCAM supplements [31,34,35].

In experimental groups of animals, deviations from the norm for cellulolytic, amylolytic, transit and pathogenic bacteria were not detected. On the other hand, a microbiota with antimicrobial activity that stimulates animal immunity, such as *Bifidobacterium* sp. and *Bacillus* sp., has been elevated [32,35].

Poultry abattoir waste is a slowly decomposing protein in the rumen and, thus, can be an economical and rich source of insoluble protein in the diet of ruminants. This means that the use of feed based on poultry processing waste for feeding ruminants has great potential as a cleaner animal feeding product [15,16,32,36,37,38,39,40]. Nevertheless, further studies are needed to assess the digestibility of nutrients, to study the productivity, and the blood of different sex and age groups of highly productive livestock in comparison with plant concentrated feeds. In this paper, the purpose of our research was to assess the digestibility and biochemical parameters of blood when growing dairy heifers of the Russian Ayrshire breed, depending on different levels of the protein concentrate Agro-Matic.

## 2. Materials and Methods

### 2.1. Animals and Experimental Design

This study is a part of the research of the Department of Feeding Animals Russian State Agrarian University–Moscow Timiryazev Agricultural Academy on the topic “Physiological justification of the effectiveness of the use of protein concentrate “Agro-Matic” in feeding dairy cattle” dedicated to the study of the effect of protein concentrate from poultry meat production waste in feeding young dairy cattle.

The entire research process was carried out at the Vologda region (Russia) on a farm belonging to Agricultural Production Cooperative Plemzavod Maysky on dairy heifers since the transition from dairy feed to solid feed. A total of 48 Ayrshire heifers were selected for the experiment and divided into 3 experimental groups (including the control group) (Table 1).

When establishing groups, origin, age, live weight at birth and at 60 days of age and health status were taken into account. Before starting the study, the animals were examined by a full-time veterinarian of the farm for the presence of diseases or abnormalities in the state of health. Before the start of feeding rapeseed cake (RC) and protein concentrate Agro-Matic (PCAM), all animals were subjected to veterinary examination to ensure that all animals were healthy.

The animals were clinically healthy and kept in the same conditions throughout the entire experiment. All animals were kept loose, with each group of heifers in a separate pen; the only exception was the period of the physiological experiment to determine the digestibility of basic nutrients. The animals were kept in a separate stall on a leash for 14 days before the experimental studies.

### 2.2. Ration Composition and Dietary Supplement

After calving, the cows were transferred to the department for lactating cows, and the calves that were candidates to enter the study were fed a daily amount of colostrum through a drencher and placed in separate pens, which were balanced by live weight and date of birth. Drinking milk and other dairy products was carried out according to one approved protocol on the farm. During training and transition to the main solid feeds, the diet was carried out with an average of 45–50 days from birth. The live birth weight in the control group receiving RC was 32.90 ± 0.53 kg, in the second group receiving RC + PCAM, 32.70 ± 0.54 kg, and the third receiving PCAM, 32.60 ± 0.48 kg, respectively. The composition of concentrates for Ayrshire heifers under the study are presented in Table 2.

The formulation of rations was carried out according to the Feed Optima program (v. 2020.8.17251) to meet the energy and nutritional needs of heifers during the growing periods. Part of the rapeseed cake in the second (RC + PCAM) and third (PCAM + RC) groups was partially replaced by protein concentrate Agro-Matic (PCAM), which consists of white lupine grains and poultry meat waste in the amount of 2.25% (group 2) and 4.5% (group 3). The diet of the heifers was balanced, in agreement with the recommendations for feeding highly productive dairy cattle, which was distributed 2 times a day with unhindered access to water. The inclusion of all feeds was carried out by distributing pre-prepared total mixed-ration (TMR) feed mixture individually for each section.

The nutritional value of daily ration of Ayrshire heifers is located in Table 3 and chemical nutritional analysis is presented in Table 4.

### 2.3. Chemical Analysis

The analysis of the chemical composition of feed and products of the balance experiment was carried out in the laboratory of the Northwest Research Institute of Dairy and Pasture Animal Husbandry named after A.S. Emelyanov which is considered as a separate division of the Vologda Scientific Center of the Russian Academy of Sciences (the village of Dairy, Vologda region). Feed samples and products of the balance experiment were taken in accordance with the ISO 6498:2012 standard “Animal feed and they were similarly subjected to chemical analysis according to the AOAC methods” [41]. The chemical composition of the crude materials was determined according to the following:

- Dry matter (AOAC 930.15);
- Organic matter (AOAC 924.05);
- Crude ash (AOAC 923.03);
- Crude protein and nitrogen (AOAC 984.13);
- Essential ether extract (crude fat) (AOCS approved procedure Am 5-04);
- Crude fiber (AOCS Ba-05 standard procedure);
- Calcium and phosphorus (ISO 27085:2009) [42].

Nitrogen in urine is nitrogen according to the Kjeldahl method (AOAC 984.13).

### 2.4. Analysis of Growth Parameters of Ayrshire Heifers

During the study, the following parameters were taken into account: daily weight gain and absolute live weight at birth at the age of 3, 6 and 10 months. The following indicators were measured during the study:

The live weight (g) was determined by a control individual weighing of heifers 1 time per month from the moment of birth.

Absolute gain (A, g) is the increase in live weight over the period of the experiment, which was determined using Equation (1):

(1) $$A=W_{1}-W_{0}$$

where *W*_1_ is the live weight of heifers at the end of the growing period (final BW, g) and *W*_0_ is the live weight of heifers at the beginning of the growing period (initial BW, g). 

The average daily gain (ADG, g) was calculated by weighing the results, which was determined using Equation (2):

(2) $$V_{t}=\frac{W_{2}-W_{1}}{t_{2}-t_{1}}$$

where *W*_2_ is the live weight of heifers at the end of the growing period (g), *W*_1_ is the live weight of heifers at the beginning of the growing period (g), *t*_2_ is the age of heifers at the end of the growing period (day) and *t*_1_ is the age of heifers at the beginning of the growing period (day).

Feed costs per 1 kg of live weight gain (kg) were calculated by dividing the amount of feed consumed over the entire period of the experiment by the live weight gain of heifers during the growing period.

### 2.5. Digestibility of Nutrients and Nitrogen Balance

The use of nutrients and the use of nitrogen in diets were established based on the results of balance experiments conducted at the age of 3 months in accordance with the methodological recommendations of the Federal Research Center for Animal Husbandry named after Academician L.K. Ernst (2016) [43]. For this experiment, 3 heifers from each group were selected, which were homogeneous in live weight and reflected the average value for the group. The animals were kept in special stalls on a leash with a plank floor. The heifers were distributed over 3 diets and fed to an individual feeder, making sure that the animals consumed only feed evenly.

To compile an average sample, animal feed, leftovers and excrement were taken every day and stored in glass jars. Feed several times a day as the feed mixture was distributed and consumed, the remains of the feed from the previous day were weighed 1 time in the morning of the next day during the entire period of the experiment to calculate nutrient intake.

Feces and urine were collected in individual tanks, from which an average sample was subsequently taken 2 times a day for chemical analysis. To preserve the secretions, a 10% solution of hydrochloric acid and toluene was added to them in the calculation of 10% HCl and 0.5% toluene by weight of the average sample. The added amount of preservatives was taken into account when determining the dry matter content [44].

At the end of the experiment, the samples were dried in a drying cabinet at a temperature of 65 °C for several days and crushed for further chemical analysis. The chemical composition of the litter was analyzed similarly to the methods presented in Section 2.2.

The digestibility coefficient (DC, %) of each nutrient in the diet was evaluated using Equation (3):

(3) $$\mathrm{DC}=\frac{\mathrm{intake}\;\mathrm{nutrient}-\mathrm{excreted}\;\mathrm{in}\;\mathrm{feces}}{\mathrm{intake}\;\mathrm{nutrient}}\times 100\%$$

### 2.6. Blood Sampling and Analysis

Throughout the experiment, blood samples (9 mL) were taken from the tail vein of heifers aged 3 and 10 months. Samples were taken from the caudal vein 3 h after morning feeding. They were collected in a test tube with coagulant activator (Zhejiang Gongdong Medical Technology Co., Ltd., Huangyan, China) to obtain blood serum. Blood serum was obtained after blood sampling and centrifugation of blood samples for 15 min, 3000× *g*, and then stored at a temperature of +4 °C to determine the biochemical parameters of blood. Biochemical blood parameters were analyzed in a certified independent veterinary laboratory (Laboratory of Animal Biochemistry and Physiology Northwest Research Institute of Dairy and Grassland Farming named after A.S. Emelyanov—A separate subdivision of the Federal State Budgetary Institution of the Russian Academy of Sciences). In the blood of calves, the following parameters were measured:

- Glucose—calorimetrically with ortho-toluidine according to Gultman in the modification of Hivarinen-Nikkel;
- Pyruvic acid—according to the modified Freedman and Haugen method;
- Non-esterified fatty acids (NEFA)—calorimetrically;
- Ketone bodies—by the iodometric method;
- Total protein—by the refractometric method;
- Urea—calorimetrically;
- Calcium spectrometric titration;
- Phosphorus—colorimetric method, reserve alkalinity according to Nevodov in Lebedev modification;
- ALT—UV-kinetic method;
- AST—dinitrophenylhydrozone method;
- Carotene (mg%)—colorimetrically.

### 2.7. Statistical Analysis

The results are expressed as the means ± standard errors. Before running the statistical analysis, data were subjected to Shapiro–Wilk and Levene tests to test the normality and homogeneity of the data. Before processing percentile data, an arcsine transformation was used. Analysis was performed on both non-transformed and transformed data. However, the results were similar in both analyses; therefore, the non-transformed data were used. Data were statistically analyzed using the statistical analysis program SPSS, 2017 [45]. One-way ANOVA followed by Duncan’s multiple range tests and Tukey’s multiple comparison tests (post-hoc test) was used to compare the means. The means were considered significant at (*p* < 0.05) of the treated groups [46].

The statistical model was as follows:

Yij = μ + Gi + Eij

where Yij is an observed value of the dependent variable; μ is a constant common to all observations; Gi is an effect due to the ith treatment.

1st = rapeseed cake (RC);

2nd = rapeseed cake (RC) and protein concentrate Agro-Matic (RC + PCAM); 

3rd = protein concentrate Agro-Matic and rapeseed cake (RC) (PCAM + RC); and Eij is a random deviation due to unexplained sources of variation.

## 3. Results

### 3.1. Indicators of Growing Performance of Ayrshire Heifers

Table 5 shows the indicators of growth dynamics of the Ayrshire heifers that received different sources of protein.

The data presented in Table 5 showed that the live weight of heifers receiving Agro-Matic protein concentrate as part of the diets exceeded (*p* < 0.05) that in the control group at 3 months of age by 3.37 kg in the second group and by 5.00 kg in the third group. The same trend was obtained at the ages of 6 and 9 months of heifer raising and differences were significant between heifers receiving Agro-Matic protein concentrate as part of the diets and the control group. Moreover, absolute body gain and average daily gain recorded significant differences between the RC + PCAM, PCAM + RC and CON groups in the different periods 1–3 and 3–6 months of age, while the differences were (*p* > 0.05) during the period of 6–9 months.

### 3.2. The Digestibility of Nutrients and the Utilization of Nitrogen in the Ration

The digestibility of nutrients of Ayrshire heifers when different levels of Agro-Matic protein concentrate were included in their diets is presented in Table 6.

Referring to the previous results obtained in Table 6, it should be noted that the use of Agro-Matic protein concentrate based on poultry meat processing waste did not have a negative effect on the digestibility of nutrients except for the digestibility coefficient of EE which decreased in the second group (RC + PCAM) in comparison with the control one. The heifers of the control group were lower than the third group in terms of digestibility of organic matter (OM) by 3.2% (*p* < 0.05), and the animals of the second group in terms of digestibility of the ether extract (EE) were lower than the control by 2.7% (*p* < 0.05), respectively. The best protein digestibility was noted in the group where the animals were fed the maximum level of high-protein concentrate Agro-Matic (PCAM), exceeding the control by 3.81%. The coefficients of digestibility of crude fiber and nitrogen-free extract did not differ significantly between the groups. 

The results showing the indicators of nitrogen balance in the body of Ayrshire heifers are presented in Table 7.

According to the obtained data on nitrogen balance at the age of 3 months, the introduction of protein concentrates Agro-Matic (PCAM) had a positive effect on nitrogen balance when compared to the control heifers. Thus, the introduction of protein concentrates into the diet contributed to a more efficient use of protein in the diet, due to a more efficient digestion of nitrogen in PCAM + RC by 8.77% (*p* < 0.05) (86.09 vs. 79.15 g) respectively.

### 3.3. Biochemical Parameters of Blood Serum in Calves and Heifers

Biochemical blood parameters of Ayrshire dairy heifers at the ages of 3 and 10 months are illustrated in Table 8 and Table 9.

According to the results obtained, it was noted that the introduction of protein concentrate (PCAM) into the compound feed in the second group had a positive effect on some biochemical blood parameters. In the body of young heifers (at the age of 3 months) in the second group, which received a minimum amount (2.25%), the total content of ketone bodies decreased from 2.20 mmol/L in the control group to 1.82 mmol/L (*p* < 0.05). However, in terms of the urea content in the ROC + PCAM group, there were higher levels relative to the control group (*p* > 0.05) and the third group (*p* < 0.05). In the group of heifers receiving the maximum level of PCAM (4.50%) in the compound feed, the urea content in the blood was significantly lower by 1.57 mmol/L, compared to the second group and the control (*p* > 0.05). The alkaline reserve of blood in young dairy heifers who consumed the highest level of protein concentrate, showed an increase in blood alkalinity of 424.67 mg%, versus 414.67 mg% in the control group. So, the increase in this indicator relative to the control group was 2.41%, respectively.

Results of blood biochemistry which were conducted at 10 months of raising are given in Table 9.

According to the results obtained for the 2nd period of the study at 10 months of age, the introduction of protein concentrate contributed to an increase in the content of β-globulins due to the introduction of PCAM. The increase in the supplemented groups RC + PCAM and PCAM + RC was 0.26 g/L and 0.28 g/L relative to the control, respectively. With respect to the phosphorus level, a similar trend was found where, in the second group (RC + PCAM), with comparison to the control, an increase of 0.18 mmol/L (*p* > 0.05) was observed, and in the third group (PCAM + RC) an increase of 0.32 mmol/L (*p* < 0.05) was observed, respectively. On the other hand, the concentration of aspartate aminotransferase in the blood of RC + PCAM heifers was lower than the control group and (28.27 vs. 39.60 U/mL × h, respectively) and the difference was significant.

## 4. Discussion

### 4.1. Indicators of Growing Growth Heifers

Due to the growth of the world’s population, there is an increase in the consumption of food, especially animal products, such as milk and meat. Dairy products are the most affordable source of animal protein for all segments of the population, which makes it more attractive to producers [1,15,40]. However, it is impossible to obtain high milk productivity and full realization of the genetic potential of animal productivity without careful planning of the breeding process, compliance with the technologies of raising, maintenance, feeding and preparation of young dairy heifers for breeding in order to obtain highly productive cows [43,47].

One of the ways to improve the live weight of growing heifers is to increase the content of non-degradable protein in the diet [48,49,50]. In our study, the use of PCAM (2.25% and 4.50%) has shown a positive effect on the growth dynamics of Ayrshire dairy heifers. The supplemented animals were significantly higher than the control group. These results are primarily due to the fact that up to 6 months the calves’ rumen does not fully function and calves mainly produce digestion in the abomasum and the small part of the gastrointestinal tract. So, some authors indicated that for the development of a fully-fledged highly productive cow from a calf, it is necessary to pay attention, first of all, to the amount of protein in the diet and its amino acid composition [47,51,52].

### 4.2. The Ration

The basis for increasing the productivity of livestock is to meet the needs of the body, firstly, in the optimal intake of nutrients from the diet, and secondly, their effective decomposition to monomers in the gastrointestinal tract and use for the needs of the body and productivity [47,53,54]. Rations containing animal proteins have a more complete digestible protein, because in ruminants, more precisely in rumen microorganisms, there are no enzymes capable of breaking them down to amino acids and subsequently to amino acids [35].

Our current studies have shown that the usage of protein concentrate based on poultry meat processing waste did not have a negative effect on the digestibility of nutrients except for the digestibility coefficient of EE which decreased in the second group (RC + PCAM) in comparison with the control one. The best protein digestibility was noted in the group where the animals were fed the maximum level of PCAM, exceeding the control by 3.81%, and the coefficients of digestibility of crude fiber did not differ between the groups. In similar studies conducted by Kamalak et al. (2005) it was shown that animal protein sources, such as poultry abattoir waste with a low ability to decompose, can be used to increase the amount of byproduct protein [55]. Kazemi-Bonchenari et al. (2017) [13] showed that poultry abattoir waste contains a greater amount of slowly decomposed protein compared to fishmeal and fried soy. In this study, it was shown that the use of poultry abattoir waste has a low digestibility of DM, OM and CP when compared to the other two feeds. The high fat content in this feed, which was found in this study to be equal to 20.5%, may be one of the supposed reasons [13]. However, according to our research, the production of protein concentrates from poultry abattoir waste and together with plant components (white lupine grain) showed a positive effect on the digestibility of organic matter, crude protein and fat in young heifers. Knaus et al. (1998) suggested that bone meal and hydrolyzed feather flour can be considered as insoluble protein sources that can improve nitrogen assimilation, nitrogen balance and nitrogen utilization efficiency in young cattle [56].

Bohnert et al. (1998) reported that the loss of nitrogen in the rumen when feeding poultry processing waste was 1.4%, nitrogen was isolated with productivity in diets containing poultry abattoir waste of 55% versus 25% for soybean meal [57]. Kim et al. (2003) showed that processing poultry flour with NaOH or an enzyme led to faster protein degradation in the rumen of dairy cows and, consequently, to a greater availability of amino acids for rumen microorganisms compared to untreated poultry flour [58]. In our studies, no reliable values were obtained for the use of nitrogen at the age of 3 months of animals. In our studies, nitrogen retention in the body of heifers was significantly higher in the groups with Agro-Matic protein concentrate, which is consistent with similar works by Kamalak et al. (2005) [55] and Knaus et al. (1998) [56].

### 4.3. Biochemical Parameters of Blood Serum in Calves and Heifers

The rearing of heifers is a key issue on dairy farms, because these animals ensure the maintenance and renewal of the herd [47,59]. Thus, heifer rearing is a necessary process on which the high productivity of cows programmed at the beginning of heifer rearing depends [51,52]. Thus, the assessment of blood metabolic profiles is necessary to monitor the health, reproduction and physiological conditions of the animal, which can prevent metabolic disorders in animals [60,61].

In our studies, no clinical signs of acidosis were registered, a decrease in feed intake or diarrhea were not observed in any of the experimental animals, and blood counts corresponded to normal values for the species. Regarding urea content at the age of 3 months, the content of the maximum level of PCAM + RC decreased (*p* < 0.05). Similar results were obtained by Vosooghi-poostindoz et al. (2014) [62] who reported that the concentration of urea nitrogen in the blood was significantly influenced (*p* < 0.05) by the level of crude protein in the diet.

It is known that ammonia released during the decomposition of food or endogenous urea in the rumen contributes to maintaining the physiological pH in the rumen [63]. In our study, the concentration of urea in plasma was lower in the group with a high content of PCAM protein than in other experimental groups, which contradicts the studies of Saro et al. (2020) [64]. The decrease in the level of urea in the experimental groups is probably attributed to the content of a high amount of bypass protein in the protein concentrate, which bypasses the rumen and splits into amino acids in the small gastrointestinal tract of heifers. This is probably a consequence of the lower uptake of ammonia in the rumen and the synthesis of urea in the liver. However, there were no differences between the experimental groups in blood glucose content, which suggests that other factors, such as differences in feed intake, could affect its level. 

However, it is necessary to note a decrease in the level of ketone bodies in the blood serum of heifers at the age of 3 months, where there was a significant decrease in the concentration of ketone bodies in the blood, which indicates a more favorable level and course of energy and lipid metabolism in animals. On the other hand, the levels of liver enzymes, albumin, total protein, glucose, calcium, phosphorus and carotene were within normal values. Despite this, the introduction of protein concentrate did not significantly affect the content of total protein or albumins in the blood during the growing period. 

The levels of globulins, phosphorus and alkaline reserve in the plasma increased as the amount of protein concentrate increased. The addition of protein concentrates at the age of 3 months from the moment of birth did not affect the activity of transaminases, which indicated that damage to hepatocytes did not occur. However, the activity of aspartate aminotransferase synthesized in the liver was changed by the interaction between the levels of protein concentrate, so at this age there was a significant decrease in its content compared to the blood content of the control group receiving only plant protein sources. The decrease in the content of aminotransferase is probably reduced due to insufficient synthesis of vitamin B_6_.

Moreover, an increase in the reserve alkalinity of the blood was noted at the beginning of raising, probably due to the intake of alkaline elements with protein concentrate and their better assimilation, which is probably indirect evidence of a reduction in the risks of subacute lactic acidosis in these animals.

## 5. Conclusions

Partial replacement of rapeseed cake with the by-product source of animal protein Agro-Matic concentrate in various amounts in the diet of Ayrshire dairy heifers showed a positive effect on growth dynamics, improved digestibility of nutrients and nitrogen retention without deterioration of the general health status of animals. Therefore, we recommend supplementation with the by-product source of animal protein Agro-Matic concentrate into the diets of heifers at an appropriate dose of 4.5% during the period of 3 to 6 months of the raising stage.

## Figures and Tables

**Table 1:** Feeding scheme and research design.

Groups	Heifers Number (*n*)	The Program of Heifers Feeding
CON	16	Basic diet (BD): 0.5 kg of grain-legume hay, 3.5 kg of grain-legume haylage and 2.0 kg of concentrate, containing rapeseed cake (RC) as the main high-protein component with a rich content of crude protein in an amount of 30% and high-protein concentrate Agro-Matic (PCAM) in an amount of 0% from weight of concentrate.
RC + PCAM	16	BD, including concentrate containing (RC) in the amount of 27.75% and PCAM in the amount of 2.25% of the feed weight as the main high-protein component with a rich content of crude protein.
PCAM + RC	16	BD, including concentrate, containing (RC) as the main high-protein component with a rich content of crude protein in the amount of 25.50% and PCAM in the amount of 4.50% from weight of concentrate.

CON = control; RC = Rapeseed cake; PCAM = protein concentrate Agro-Matic.

**Table 2:** Composition of concentrates for heifers, %.

Ingredients	Type of Protein Feeds		
	CON	RC + PCAM	PCAM + RC
Peeled barley	38.15	38.15	38.15
Herbal meal	3.50	3.50	3.50
Extruded peas	12.60	12.60	12.60
Sunflower oil cake	9.80	9.80	9.80
Rapeseed oil cake	30.00	27.75	25.50
Protein concentrate Agro-Matic (PCAM)	-	2.25	4.50
Fodder yeast	3.50	3.50	3.50
Sodium chloride salt	0.35	0.35	0.35
Monocalcium phosphate	0.70	0.70	0.70
Limestone powder	0.70	0.70	0.70
Vitamin-trace mineral premixes	0.70	0.70	0.70

CON = control; RC = Rapeseed cake; PCAM = protein concentrate Agro-Matic.

**Table 3:** Nutritional value of daily ration of Ayrshire heifer.

Indicators	UM	Type of Protein Feeds		
		CON	RC + PCAM	PCAM + RC
Metabolizable energy	MJ	38.0	38.1	38.2
Crude protein	g	673.8	685.2	696.7
Digestible protein	g	512.3	523.1	533.9
Rumen degradable protein	g	431.5	432.9	434.2
Rumen ungradable protein	g	241.6	251.8	262.0
Crude fiber	g	778.4	774.5	770.6
Nonstructural carbohydrates	g	661.3	663.0	664.9
Crude fat	g	159.3	160.2	161.0
Calcium	g	26.9	28.6	30.3
Phosphorus	g	16.8	17.0	17.1

CON = control; RC = Rapeseed cake; PCAM = protein concentrate Agro-Matic. UM—Units of measurement. DM—Dry matter. CP—crude protein. DP—digestible protein. RDP—rumen degradable protein. RUP—rumen ungradable protein. NSC—Nonstructural carbohydrates.

**Table 4:** Analysis of the nutritional value of the diet of Ayrshire heifers.

Indicators	UM	Type of Protein Feeds		
		CON	RC + PCAM	PCAM + RC
Metabolizable energy	MJ per 1 kg DM	10.1	10.1	10.1
Dry matter	%	62.9	62.9	62.9
Crude protein	% per DM	18	18	18
RUP-to-UDP ratio	unit	1.79(64:36)	1.72(63:37)	1.66(62:38)
NSC-to-DP ratio	unit	1.29	1.27	1.25
Crude fiber	% per DM	20.6	20.5	20.4
Essential extract	% per DM	4.2	4.2	4.3

CON = control; RC = Rapeseed cake; PCAM = protein concentrate Agro-Matic. UM—Units of measurement. DM—Dry matter. CP—crude protein. DP—digestible protein. RDP—rumen degradable protein. RUP—rumen ungradable protein. NSC—Nonstructural carbohydrates.

**Table 5:** Growth dynamics indicators of growing Ayrshire heifers.

Indicators	Type of Protein Feeds			*p*-Value
	CON	RC + PCAM	PCAM + RC	
Live weight of heifers, kg				
At birth	32.94 ± 0.37	32.69 ± 0.42	32.63 ± 0.39	0.837
2 months	55.63 ± 0.52	55.56 ± 0.48	55.81 ± 0.47	0.933
3 months	103.13 ± 0.68 ^b^	106.50 ± 0.75 ^a^	108.13 ± 0.59 ^a^	0.001
6 months	174.63 ± 1.18 ^b^	179.06 ± 1.70 ^ab^	182.75 ± 1.12 ^a^	0.001
9 months	234.50 ± 1.11 ^b^	242.38 ± 3.25 ^a^	244.25 ± 1.44 ^a^	0.006
Absolute body gain, kg				
1–3 months	70.19 ± 0.43 ^b^	73.81 ± 0.63 ^a^	75.50 ± 0.56 ^a^	0.001
3–6 months	119.00 ± 1.08 ^b^	123.50 ± 1.61 ^a^	126.94 ± 1.10 ^a^	0.001
6–9 months	131.38 ± 0.93	135.88 ± 3.24	136.13 ± 1.52	0.219
Average daily gain, g				
1–3 months	779.86 ± 4.78 ^b^	820.14 ± 6.97 ^a^	838.89 ± 6.17 ^a^	0.001
3–6 months	991.67 ± 9.03 ^b^	1029.17 ± 13.44 ^a^	1057.81 ± 9.14 ^a^	0.001
6–9 months	729.86 ± 5.14	754.86 ± 17.98	756.25 ± 8.45	0.219

CON = control; RC = Rapeseed cake; PCAM = protein concentrate Agro-Matic. Values are expressed as means ± SE. Means within the same row with different superscripts are significantly different (*p* < 0.05).

**Table 6:** Digestibility of nutrients in Ayrshire heifers at the age of 3 months.

Indicators, %	Type of Protein Feeds			*p*-Value
	CON	RC + PCAM	PCAM + RC	
DM	64.4 ± 1.42	65.6 ± 0.43	67.4 ± 0.68	0.127
OM	66.7 ± 0.58 ^b^	68.4 ± 0.63 ^ab^	69.9 ± 0.80 ^a^	0.047
CP	73.42 ± 0.46 ^b^	74.06 ± 0.72 ^ab^	77.23 ± 0.48 ^a^	0.006
EE	69.7 ± 0.37 ^a^	67.0 ± 0.45 ^b^	70.8 ± 0.53 ^a^	0.012
CF	55.3 ± 0.74	56.5 ± 0.58	57.4 ± 0.70	0.395
NFE	70.7 ± 0.63	72.6 ± 0.77	74.2 ± 1.22	0.348

CON = control; RC = Rapeseed cake; PCAM = protein concentrate Agro-Matic. Values are expressed as means ± SE. Means within the same row with different superscripts are significantly different (*p* < 0.05). DM—dry matter, OM—organic matter, EE—essential extract, CP—crude protein, CF—crude fiber, NFE—nitrogen-free extractive substances.

**Table 7:** Indicators of nitrogen balance in the heifers at the age of 3 months.

Indicators	Type of Protein Feeds			*p*-Value
	CON	RC + PCAM	PCAM + RC	
Intake nitrogen, g	107.80 ± 1.49	109.64 ± 0.76	111.47 ± 0.66	0.118
Excreted in feces, g	27.65 ± 1.46	28.47 ± 0.79	25.39 ± 0.67	0.172
Digested nitrogen, g	79.15 ± 1.28 ^b^	81.17 ± 0.95 ^a^	86.09 ± 0.10 ^a^	0.005
Excreted in urine, g	49.17 ± 1.02	49.54 ± 0.86	51.06 ± 0.52	0.302
Nitrogen retention, g	29.98 ± 1.94	31.63 ± 0.19	35.03 ± 0.48	0.054
from intaked, %	27.78 ± 1.57	28.88 ± 0.31	31.47 ± 0.24	0.075
from digested, %	37.86 ± 1.89	38.98 ± 0.37	40.69 ± 0.58	0.296

CON = control; RC = Rapeseed cake; PCAM = protein concentrate Agro-Matic. Values are expressed as means ± SE. Means within the same row with different superscripts are significantly different (*p* < 0.05).

**Table 8:** Biochemical parameters of blood serum in Ayrshire heifers at the age of 3 months.

Indicators	Measurement Units	Type of Protein Feeds			*p*-Value
		CON	RC + PCAM	PCAM + RC	
Glucose	mmol/L	4.28 ± 0.24	4.34 ± 0.22	4.33 ± 0.21	0.980
Ketone bodies	mmol/L	2.20 ± 0.05 ^a^	1.82 ± 0.11 ^b^	2.15 ± 0.03 ^a^	0.019
Non-esterified fatty acids	mEq/ml	0.19 ± 0.01	0.19 ± 0.04	0.23 ± 0.01	0.523
Pyruvic acid	µmol/L	112.10 ± 8.98	115.90 ± 6.12	131.10 ± 6.87	0.239
Total protein	g/L	65.07 ± 0.97	65.83 ± 2.04	64.53 ± 0.77	0.805
Albumin	g/L	29.27 ± 1.78	32.83 ± 1.40	31.70 ± 0.75	0.251
α1-globulin	g/L	5.43 ± 0.44	5.33 ± 0.43	5.67 ± 0.23	0.823
α2-globulin	g/L	5.30 ± 0.89	5.33 ± 0.43	6.17 ± 0.27	0.538
β-globulin	g/L	6.87 ± 0.13	6.43 ± 0.17	7.13 ± 0.48	0.331
γ-globulin	g/L	13.22 ± 5.91	15.93 ± 1.27	14.33 ± 0.73	0.864
Protein index	g/L	0.82 ± 0.07	0.99 ± 0.03	0.97 ± 0.02	0.075
Urea	mmol/L	5.72 ± 0.54 ^ab^	6.62 ± 0.11 ^a^	5.05 ± 0.06 ^b^	0.038
Calcium	mmol/L	2.57 ± 0.12	2.54 ± 0.26	2.48 ± 0.09	0.934
Phosphorus	mmol/L	2.04 ± 0.13	2.22 ± 0.01	2.13 ± 0.04	0.341
Calcium–phosphorus ratio	unit	1.65 ± 0.17	1.48 ± 0.15	1.50 ± 0.08	0.650
Alkalinity	mg%	414.67 ± 1.33 ^b^	420.00 ± 2.31 ^ab^	424.67 ± 2.91 ^a^	0.048
Aspartate aminotransferase	U/mL × h	31.50	30.4 ± 3.53	25.03 ± 2.48	0.416
Alanine aminotransferase	U/mL × h	28.50 ± 10.63	34.97 ± 8.83	20.97 ± 3.72	0.525
Carotene	mg%	0.34 ± 0.02 ^b^	0.41 ± 0.01 ^b^	0.48 ± 0.02 ^a^	0.003

CON = control; RC = Rapeseed cake; PCAM = protein concentrate Agro-Matic. Values are expressed as means ± SE. Means within the same row with different superscripts are significantly different (*p* < 0.05).

**Table 9:** Biochemical parameters of blood serum in calves at the age of 10 months.

Indicators	Measurement Units	Type of Protein Feeds			*p*-Value
		CON	RC + PCAM	PCAM + RC	
Glucose	mmol/L	4.35 ± 0.28	4.16 ± 0.28	4.56 ± 0.34	0.659
Ketone bodies	mmol/L	1.53 ± 0.04	1.58 ± 0.05	1.58 ± 0.16	0.933
Non-esterified fatty acids	mEq/ml	0.86 ± 0.26	0.34 ± 0.09	0.55 ± 0.12	0.175
Pyruvic acid	µmol/L	109.44 ± 2.28	110.96 ± 8.76	111.34 ± 8.89	0.981
Total protein	g/L	68.60 ± 1.69	68.00 ± 0.53	66.83 ± 1.03	0.590
Albumin	g/L	22.80 ± 2.88	30.47 ± 0.13	26.27 ± 1.82	0.086
α1-globulin	g/L	0.86 ± 0.09	0.59 ± 0.06	0.70 ± 0.05	0.082
α2-globulin	g/L	0.96 ± 0.11	0.67 ± 0.02	0.84 ± 0.05	0.072
β-globulin	g/L	0.63 ± 0.02 ^b^	0.89 ± 0.02 ^a^	0.91 ± 0.06 ^a^	0.003
γ-globulin	g/L	1.84 ± 0.15	1.87 ± 0.09	1.63 ± 0.06	0.263
Protein index	g/L	0.51 ± 0.11	0.81 ± 0.01	0.65 ± 0.06	0.064
Urea	mmol/L	3.22 ± 0.25	3.53 ± 0.10	3.63 ± 0.25	0.422
Calcium	mmol/L	2.28 ± 0.14	2.16 ± 0.12	2.20 ± 0.07	0.752
Phosphorus	mmol/L	1.55 ± 0.08 ^b^	1.73 ± 0.08 ^ab^	1.87 ± 0.06 ^a^	0.049
Calcium–phosphorus ratio	unit	1.58 ± 0.11	1.62 ± 0.11	1.84 ± 0.10	0.242
Alkalinity	mg%	484.00 ± 8.00	484.00 ± 8.33	490.67 ± 11.39	0.848
Aspartate aminotransferase	U/mL × h	39.60 ± 0.92 ^a^	28.27 ± 3.35 ^b^	34.73 ± 0.95 ^ab^	0.024
Alanine aminotransferase	U/mL × h	40.33 ± 6.47	43.63 ± 3.23	29.60 ± 10.80	0.436
Carotene	mg%	0.41 ± 0.02	0.44 ± 0.02	0.48 ± 0.01	0.101

CON = control; RC = Rapeseed cake; PCAM = protein concentrate Agro-Matic. Values are expressed as means ± SE. Means within the same row with different superscripts are significantly different (*p* < 0.05).

## Data Availability

The crude data supporting the conclusions of this article will be made available by the authors, without undue reservation. The data presented in this study are available upon request from the corresponding author.
